# Approaches in Adult Glioblastoma Treatment: A Systematic Review of Emerging Therapies

**DOI:** 10.7759/cureus.67856

**Published:** 2024-08-26

**Authors:** Billy McBenedict, Wilhelmina N Hauwanga, Anna Pogodina, Gurinder Singh, Anusha Thomas, Abdullah Mohammed Abdullah Ibrahim, Chukwuwike Johnny, Bruno Lima Pessôa

**Affiliations:** 1 Neurosurgery, Fluminense Federal University, Niterói, BRA; 2 Family Medicine, Faculty of Medicine, Federal University of the State of Rio de Janeiro, Rio de Janeiro, BRA; 3 Faculty of Medicine, University of Buckingham, Buckingham, GBR; 4 Medical Sciences, Specialized University of the Americas, Panama, PAN; 5 Neurology, Christian Medical College, Ludhiana, IND; 6 Internal Medicine, Faculty of Medicine, University of Gezira, Wad Madani, SDN; 7 Family Medicine, Lifepoint Medical Centre, Abuja, NGA

**Keywords:** immunotherapy, drug delivery systems, blood-brain barrier, emerging therapies, glioblastoma

## Abstract

Glioblastoma (GB) is the most common and aggressive primary brain tumor in adults, characterized by complex genetic changes and a poor prognosis. Current standard therapies, including surgery, chemotherapy, and radiotherapy, have limited effectiveness. Emerging therapeutic strategies aim to address the high recurrence rate and improve outcomes by targeting glioblastoma stem cells (GSCs), the blood-brain barrier, and utilizing advanced drug delivery systems. This systematic review followed the Preferred Reporting Items for Systematic Reviews and Meta-Analysis (PRISMA) guidelines. An electronic search was conducted across several databases, including PubMed, Embase, Scopus, Web of Science, and Cochrane, covering studies published from January 2019 to May 2024. The inclusion criteria encompassed primary research studies in English focusing on emerging therapies for treating GB in adults. Eligible studies included experimental and observational studies. Only peer-reviewed journal articles were considered. Exclusion criteria included non-human studies, pediatric studies, non-peer-reviewed articles, systematic reviews, case reports, conference abstracts, and editorials. The search identified 755 articles and, finally, 24 of them met the inclusion criteria. The key findings highlight various promising therapies. Despite advances in treatment approaches, the complexity and heterogeneity of GB necessitate ongoing research to optimize these innovative strategies. The study has limitations that should be considered. The inclusion of only English-language articles may introduce language bias, and the focus on peer-reviewed articles could exclude valuable data from non-peer-reviewed sources. Heterogeneity among studies, particularly in sample sizes and designs, complicates comparison and synthesis, while the reliance on preclinical models limits generalizability to clinical practice. Nonetheless, this review provides a comprehensive overview of the emerging therapies that hold promise for improving patient outcomes in GB treatment.

## Introduction and background

Glioblastoma (GB) is the most common and aggressive primary brain tumor in adults, accounting for a significant proportion of malignant primary tumors of the brain and central nervous system (CNS). Its characteristic is marked by complex genetic changes that affect several signaling pathways related to cell proliferation, survival, invasion, adaptation to hypoxia, neoangiogenesis, and resistance to apoptosis. Emerging therapeutic strategies aim to address the high recurrence rate and poor prognosis of GB. These include immunotherapy, targeting glioblastoma stem cells (GSCs), research on the blood-brain barrier (BBB), and advanced drug delivery systems such as polymer-based systems and T cell engineering. Additionally, the tumor-treating fields (TTFs) have shown promise in prolonging overall survival when used with radiotherapy and chemotherapy, increasing the median survival period from 16 to 20.9 months [1]. Other innovative approaches under investigation include therapeutic vaccines, stem cell therapy, and focused ultrasound (FUS) [2]. Despite advances in treatment, GB remains largely intractable, with a median overall survival of about 15 months after diagnosis and only 5.5% of patients surviving beyond five years [3].

Advances in molecular pathogenesis have allowed a more precise characterization of the GB microenvironment, encompassing genomic, epigenomic, transcriptomic, and proteomic analyses and their interactions with the immune system. Intratumoral and intertumoral heterogeneity is pronounced, varying in cell types, sizes, mitotic activity, cell density, calcification, vascularization, and necrosis [1]. This diversity, combined with the infiltrative nature of the tumor and the protective environment of the CNS, significantly reduces the effectiveness of conventional therapies. Furthermore, recurrent tumors often have distinct molecular profiles, which limits the utility of initial biopsies for treating recurrent disease, highlighting the urgent need for innovative therapeutic strategies to improve patient outcomes [1].

Despite a relatively simple mutagenomic profile, some canonical mutations drive oncogenesis in GB, which is notably infiltrative and migratory, protected by the BBB in an organ that hardly supports inflammation and immunity. One of the few innovations that has improved survival in GB is the use of low-intensity alternating electrical fields, known as TTFs, which have increased median survival from 16 to 21 months and raised the survival rate from five years from 5% to 13% in exploratory studies [4,5]. However, many innovative treatments have not yet been able to significantly prolong survival in GB, revealing a critical gap in the effectiveness of emerging therapies. So an efficient approach is required to identify and evaluate new medicines in the early stages of development of the disease. 

Advances in understanding the BBB and the blood-tumor barrier are essential for developing new technologies that improve anticancer drug delivery, with promising approaches including stem cell therapy, polymer-based systems, engineering of T cells, therapeutic vaccines, and FUS, aiming to improve the penetration of drugs through the BBB [6]. Furthermore, oncolytic viruses emerge as promising treatments using recombinant DNA technologies to selectively destroy GB cells, with clinical trials underway to evaluate their potential in combination with other immunotherapies [5]. Re-irradiation is also shown to be a viable approach for patients with recurrent GB, although questions remain about the efficacy and toxicity of a second course of radiation [7].

Emerging therapies, focused on tumor heterogeneity and mutations, are crucial for the development of individualized treatments, requiring further clinical trials to verify the full efficacy of immunotherapy and understand the role of GSCs in chemoresistance [2]. Hence, this review aims to highlight emerging therapies, exploring their potential to overcome the limitations of current treatments.

## Review

Materials and methods

The systematic review adhered to the principles outlined in the Preferred Reporting Items for Systematic Reviews and Meta-Analyses (PRISMA) guidelines for the organization and reporting of its results [8]. An electronic search was conducted across several research databases, including PubMed, Embase, Scopus, Web of Science, and Cochrane (Table 1). These databases were accessed on May 14, 2024. The search covered the period from January 2019 to May 2024.

**Table 1:** Summary of the search strategy employed for searching the databases

Database	Search strategy	Filters used
PubMed	(("glioblastoma"[Title/Abstract] OR "glioblastoma multiforme"[Title/Abstract] OR "GBM"[Title/Abstract] OR "astrocytoma grade iv"[Title/Abstract] OR "Grade IV Astrocytomas"[Title/Abstract] OR "Giant Cell Glioblastoma"[Title/Abstract]) AND ("emerging therapies"[Title/Abstract] OR "novel therapies"[Title/Abstract] OR "new treatments"[Title/Abstract]))	Humans only, English language, exclude preprints, filter years 2019-2024
Embase	('glioblastoma':ab,ti OR 'glioblastoma multiforme':ab,ti OR 'gbm':ab,ti OR 'astrocytoma grade iv':ab,ti OR 'grade iv astrocytomas':ab,ti OR 'giant cell glioblastoma':ab,ti) AND ('emerging therapies':ab,ti OR 'novel therapies':ab,ti OR 'new treatments':ab,ti)	Humans only, English language, filter years 2019-2024
Scopus	(TITLE-ABS-KEY ("glioblastoma" OR "glioblastoma multiforme" OR "GBM" OR "astrocytoma grade iv" OR "Grade IV Astrocytomas" OR "Giant Cell Glioblastoma") AND TITLE-ABS-KEY ("emerging therapies" OR "novel therapies" OR "new treatments") )	Humans only, English language, filter years 2019-2024
Web of Science	(AB=("glioblastoma" or "glioblastoma multiforme" or "GBM" or "Astrocytoma, Grade IV" or "Grade IV Astrocytomas" or "Giant Cell Glioblastoma")) AND AB=("emerging therapies" or "novel therapies" or "new treatments")	Humans only, English language, filter years 2019-2024
Cochrane	("glioblastoma" OR "glioblastoma multiforme" OR "GBM" OR "astrocytoma grade iv" OR "Grade IV Astrocytomas" OR "Giant Cell Glioblastoma"):ti,ab,kw AND ("emerging therapy" or "emerging therapies" or "emerging treatment" or "emerging treatments" OR "novel therapy" or "novel therapies" or "novel treatment" or "novel treatments" OR "new therapy" or "new therapies" or "new treatment" or "new treatments" OR "contemporary therapy" or "contemporary therapies" or "contemporary treatment" or "contemporary treatments" OR "modern therapy" or "modern therapies" or "modern treatment" or "modern treatments"):ti,ab,kw	Humans only, English language, filter years 2019-2024

Inclusion and exclusion criteria

The inclusion criteria encompassed studies focusing on emerging therapies for treating glioblastoma in adult humans. The eligible study designs included primary research published in English, comprising experimental studies (randomized controlled trials) and observational studies (cohort studies, case-control studies, and cross-sectional studies). Clinical cases were included only if they involved more than 10 participants. The review targeted studies on treatments such as immunotherapy, targeted therapy, nanotechnology-based therapies, tumor-treating fields, stem cell-based therapies, metabolic targeting, and epigenetic therapies. Only peer-reviewed journal articles in English were considered. The exclusion criteria omitted non-human or pediatric studies, articles unrelated to emerging GB therapies, non-peer-reviewed articles, systematic reviews, case reports, conference abstracts, and editorials.

Results

**Figure 1:**
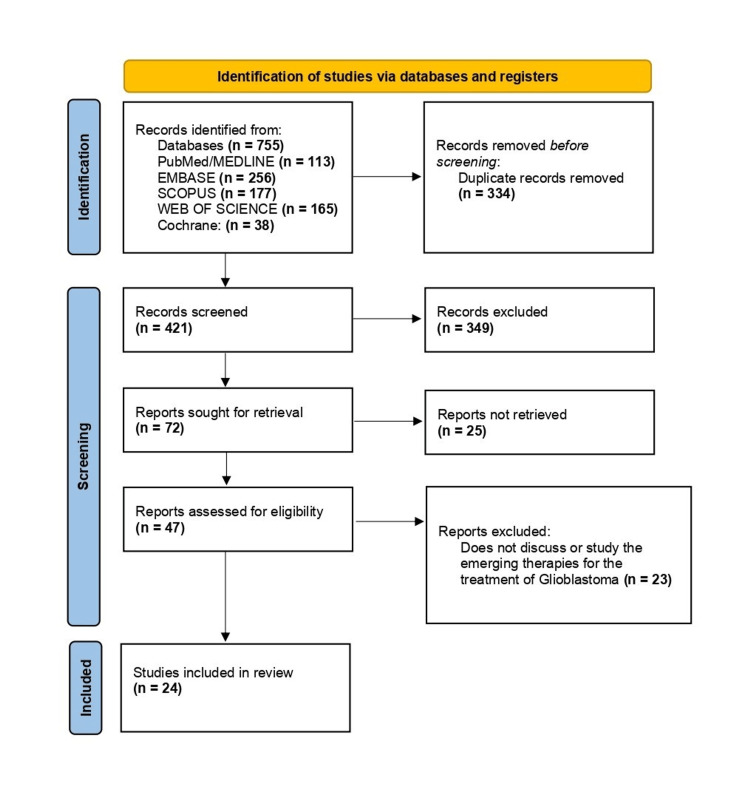
Preferred Reporting Items for Systematic Reviews and Meta-Analyses (PRISMA) flow diagram indicating the steps taken to filter the articles for this review

Through our search strategy, we identified a total of 755 articles (Figure 1), comprising 113 from PubMed/Medline, 256 from Embase, 177 from Scopus, 165 from Web of Science, and 38 from Cochrane. Filters were applied based on the inclusion/exclusion criteria. The articles were transferred to an Excel sheet, where 334 duplicates were manually removed, resulting in 421 articles. These 421 articles were further scrutinized based on their titles and abstracts, leading to the disqualification of 349, leaving 72 articles. Full texts for 25 articles could not be retrieved, leaving us with 47 papers for eligibility assessment. After a thorough full-text review, 23 papers were excluded, resulting in 24 articles being included in the final review (Table 2). Data screening was independently conducted by two review authors, with a third reviewer consulted in cases of disagreement. Notably, no automated tools were utilized in this process.

**Table 2:** Summary of studies included in this review, along with their respective demographics and key findings. GB = glioblastoma; NCAD = N-cadherin; GSCs = glioblastoma stem cells; EGFR = epidermal growth factor receptor; EC50 = half-maximal effective concentration; CSCs = cancer stem cells; MAPK = mitogen-activated protein kinase; VEGF = vascular endothelial growth factor; IL = interleukin; CD70 = cluster of differentiation 70; CAR-T: chimeric antigen receptor T; FDA = Food and Drug Administration; ILT2 = Ig-like transcript 2; NK = natural killer; IFN-γ = interferon-gamma; TMZ = temozolomide; CMV = cytomegalovirus; mOS = median overall survival; MGMT = O-6-methylguanine-DNA methyltransferase; DCVax-L = lysate-loaded dendritic cell vaccination; DFMO = difluoromethylornithine; SoC = standard of care; PPV = personalized peptide vaccination; ITK = T-cell kinase; OVT = oncolytic viral therapy; TTFs = tumor-treating fields; QoL = quality of life; ACD = asymmetric cell division.

Author	Demographics	Key findings
Smits et al. [9]	The study involved a total of 50 patients diagnosed with GB, including 29 males and 21 females	Compound 15, an NCAD antagonist, significantly reduces GB cell viability in preclinical models, from 82.6% to 16.8% over 30 days, by disrupting cell-cell adhesion.
Maruyama et al. [10]	Age and gender not explicitly mentioned in the provided text	Targeted therapies focusing on GSCs show promise, with tamoxifen demonstrating significant cytotoxicity against drug-resistant P4E8 cells, indicating potential for overcoming GB treatment resistance.
El Khayari et al. [11]	The study included a total population of 1152 patients, with a gender distribution of 54% male and 46% female	Emerging GB therapies target genetic and metabolic alterations, with inhibitors like AG-881 and CB-839 showing promise, and EGFR inhibitors extensively used; monitoring includes genetic and metabolic profiling.
McCabe et al. [12]	60 participants, evenly split by gender with 30 males and 30 females, aged between 25 and 65 years	Nanoparticle-based therapies improve GB targeting, with silica-coated gold nanoparticles showing selective uptake in proliferating spheroid layers but limited penetration due to their size.
Petővári et al. [13]	18 patients with high-grade glioma, consisting of six males and 12 females. The age distribution was 12 patients younger than 65 years and six patients aged 65 or older​	Combining rapamycin with metabolic inhibitors like doxycycline and etomoxir significantly inhibits GB cell proliferation, reducing viability to 66% and 70%, respectively, highlighting the potential of multi-pathway targeting.
Valero et al. [14]	Age and gender not explicitly mentioned in the provided text	The study identified eCF324 as a potent PI3K/mTOR inhibitor for GB, with EC50 values of 13 nM and 10 nM in U87 and T98 cell lines, respectively, and demonstrated strong synergistic effects with GDC0941, showing superior potency and high specificity for glioma cells.
Kalimuthu et al. [15]	Age and gender not explicitly mentioned in the provided text.	The tyrosine kinase inhibitor K905-0266 significantly reduces GB cell viability, induces apoptosis, inhibits the MAPK pathway, and decreases sphere formation in CSCs.
Zhang et al. [16]	Age and gender not explicitly mentioned in the provided text.	The novel oncolytic adenovirus Ad5-Ki67/IL-15 selectively targets GB cells, significantly increases IL-15 expression, reduces cell viability, and demonstrates strong anti-angiogenic properties by decreasing VEGF secretion.
Hedna et al. [17]	Age and gender not explicitly mentioned in the provided text	The 2-aminothiazole-fused flavonoid hybrids effectively target Tau protein in glioblastoma, inhibiting fibrillation, enhancing mitochondrial fission, and reducing cell migration.
Seyfrid et al. [18]	Age and gender not explicitly mentioned in the provided text	CD70-directed CAR-T cells show promise in treating GB by targeting CD70, which is overexpressed in GB, reducing tumorigenicity and enhancing survival rates in animal models.
Roddy et al. [19]	11 patients, including eight males and three females, with an average age of 49 years	GB therapies show limited efficacy, with transcriptome profiling identifying 24 genes for potential immunotherapy targets and FDA-approved drugs like rosiglitazone as promising for treating recurrence.
Vargas et al. [20]	Age and gender not explicitly mentioned in the provided text	Targeting the P2Y12 receptor with ticagrelor significantly reduces GB cell viability and proliferation, decreasing cell viability by 34.2% in U251 and 44.2% in LS12 cell lines, and increasing autophagy.
Lorenzo-Herrero et al. [21]	Age and gender not explicitly mentioned in the provided text	Blocking ILT2 enhances immune responses in GB, increasing NK cell cytotoxicity and IFN-γ production, with synergy observed when combined with temozolomide, improving tumor cell elimination.
Fleurence et al. [22]	Age and gender not explicitly mentioned in the provided text	Combining anti-O-acetyl GD2 antibody 8B6 with temozolomide significantly reduces GB tumor volume (26.8 mm³ vs. 265.2 mm³ with TMZ alone), targeting chemoresistant GSCs.
Batich et al. [23]	23 patients (67% male, median age 55)	CMV-specific dendritic cell vaccines significantly improve GB survival, with 33-36% five-year survival rates and mOS of 37.7 months, compared to 14.6-20.9 months with standard treatments.
Sim et al. [24]	Median age of participants was 61 years, and 70% were male.	Combining veliparib with temozolomide and radiotherapy offers limited benefit for GB with unmethylated MGMT promoter, showing similar mOS to standard treatment (12.7 vs. 12.8 months).
Liau et al. [25]	The median age of participants was 56 years, and 61.0% were male	DCVax-L significantly extends GB patient survival, with mOS of 19.3 months for newly diagnosed and 13.2 months for recurrent cases, showing a favorable safety profile.
Riviere-Cazaux et al. [26]	Patients aged 18 years and older	Combining DFMO and AMXT-1501 for GB shows potential, significantly altering tumor metabolites, with a 126-fold increase in guanidinoacetate, highlighting targeted metabolic therapy's importance.
Houweling et al. [27]	Age and gender are not explicitly mentioned in the provided text	Multi-targeted therapies for GB show promise, with combinations like Venetoclax and AZD5991 demonstrating strong synergy; however, close monitoring is essential due to potential toxicities.
Barkhoudarian et al. [28]	Age and gender are not explicitly mentioned in the provided text	The Voyager system for recurrent GB is safe and promising, showing mOS of seven months alone and nine months with chemotherapy, with no serious device-related adverse events.
Narita et al. [29]	The study included four groups: Voyager only (32 patients, mean age 55.97, 41% female), Voyager + SoC (43 patients, mean age 53.09, 40% female), Voyager + SoC 1st and 2nd recurrences (32 patients, mean age 51.91, 41% female), and Voyager + SoC 3rd and 4th recurrences (11 patients, mean age 56.55, 36% female).	PPV for recurrent GB showed limited effectiveness, with mOS of 8.4 months for PPV versus 8.0 months for placebo, failing to meet primary endpoints.
Lu et al. [30]	The study included 88 GB patients: 58 in the ITK-1 group (median age 52.5, 37 males, 21 females) and 30 in the placebo group (median age 59 years; 19 males, 11 females).	OVT for GB shows promise, with median progression-free survival of 3 months and overall survival of 15 months, enhancing treatment efficacy through various mechanisms.
Koguchi et al. [31]	Median enrollment target of 36 patients (range 13–108).	BMP4 therapy for GB induces ACD, reduces GSCs, decreases CD133 expression, and enhances chemotherapy effectiveness by promoting neural differentiation without affecting cell viability.
Onken et al. [32]	30 patients. The mean age was 50 years (median 52 years, range 36–64), with a male predominance of 67%. The total population in the control group was 27 patients​	TTF therapy for GB enhances emotional functioning and reduces insomnia, but 71% of patients face significant activity restrictions, with 67% willing to reuse it; monitoring includes standardized QoL assessments.

Study quality and bias assessment

The quality of the articles were assessed using the Joanna Briggs Institute (JBI) Critical Appraisal Tools (Table 3). The JBI appraisal tool includes questions that allow for the assessment of the quality of articles in a systematic review. In addition, it allows for the identification of biases, errors, or flaws in the study methodologies, results and/or conclusions drawn. Hence, this process also led to the removal of poor quality articles. All studies included in the analysis focused on a clearly defined issue regarding glioblastoma treatment. Studies recruited participants in a clearly defined manner or clearly stated how samples were obtained (Table 3). However, numerous studies displayed significant differences in gender representation among participants.

**Table 3:** The quality assessement using the JBI Critical Appraisal Tool Y= Yes, N= No, NC= Not clear, N/A= Not Applicable Joanna Briggs Institute (JBI) appraisal checklist is based on nine items and each item is assessed by scoring (yes = 1),  (no = 0), and (not clear or not applicable = 0). The total score obtained for each individual study was presented as percentages and each study was categorized according to diﬀerent levels of risk of bias (high risk of bias if 20–50% items scored yes, moderate risk of bias if 50–80% items scored yes, and low risk of bias if 80–100% items scored yes as per the JBI checklist.

Checklist question	[9]	[10]	[11]	[12]	[13]	[14]	[15]	[16]	[17]	[18]	[19]	[20]	[21]	[22]	[23]	[24]	[25]	[26]	[27]	[28]	[29]	[30]	[31]	[32]
Were there clear criteria for inclusion in the case series?	Y	Y	Y	Y	Y	Y	Y	Y	Y	Y	Y	Y	Y	Y	Y	Y	Y	Y	Y	Y	Y	Y	Y	Y
Was the condition measured in a standard, reliable way for all participants included in the case series?	Y	Y	Y	Y	Y	Y	Y	Y	Y	Y	Y	Y	Y	Y	Y	Y	Y	Y	Y	Y	Y	Y	Y	Y
Were valid methods used for the identification of the condition for all participants included in the case series?	Y	Y	Y	Y	Y	Y	Y	Y	Y	Y	Y	Y	Y	Y	Y	Y	Y	Y	Y	Y	Y	Y	Y	Y
Did the case series have the consecutive inclusion of participants?	Y	N/A	Y	Y	Y	N/A	N/A	N/A	N/A	N/A	Y	N/A	N/A	Y	Y	Y	Y	Y	Y	Y	Y	Y	Y	Y
Did the case series have a complete inclusion of participants?	Y	Y	Y	Y	Y	Y	Y	Y	Y	Y	Y	Y	Y	Y	Y	Y	Y	Y	Y	Y	Y	Y	Y	Y
Was there clear reporting of the demographics of the participants in the study?	Y	N/A	Y	Y	Y	N/A	N/A	N/A	N/A	N/A	Y	Y	Y	Y	Y	Y	Y	Y	Y	Y	Y	Y	Y	Y
Were the outcomes or follow-up results of cases clearly reported?	Y	Y	Y	Y	Y	Y	Y	Y	Y	Y	Y	Y	Y	Y	Y	Y	Y	Y	Y	Y	Y	Y	Y	Y
Was there clear reporting of the presenting sites’ or clinics’ demographic information?	N/C	Y	Y	Y	Y	Y	Y	Y	Y	Y	Y	Y	Y	Y	Y	Y	Y	Y	Y	Y	Y	N/C	Y	Y
Was the statistical analysis adequate?	Y	Y	Y	Y	Y	Y	Y	Y	Y	Y	Y	Y	Y	Y	Y	Y	Y	Y	Y	Y	Y	Y	Y	Y
Score	8/9 (89%)	7/9 (78%)	9/9 (100%)	9/9 (100%)	9/9 (100%)	7/9 (78%)	7/9 (78%)	7/9 (78%)	7/9 (78%)	7/9 (78%)	9/9 (100%)	8/9 (89%)	8/9 (89%)	9/9 (100%)	9/9 (100%)	9/9 (100%)	9/9 (100%)	9/9 (100%)	9/9 (100%)	9/9 (100%)	9/9 (100%)	8/9 (89%)	9/9 (100%)	9/9 (100%)
Risk of bias	Low	Moderate	Low	Low	Low	Moderate	Moderate	Moderate	Moderate	Moderate	Low	Low	Low	Low	Low	Low	Low	Low	Low	Low	Low	Low	Low	Low

Discussion

N-cadherin antagonist: Compound 15

A novel approach for treating GB involves using an N-cadherin (NCAD) antagonist, Compound 15, which has shown significant efficacy in preclinical models [9]. In a 3D bioprinted model, Compound 15 was effective in inducing cell death and preventing spheroid formation. Cell viability in treated cultures decreased substantially over 30 days, with measurements at 5 mM showing viability dropping from 82.6% ± 14.8% on day 16 to 16.8% ± 1.0% on day 30 [9]​. Higher concentrations (10 mM and 25 mM) resulted in even more rapid declines, with viability reaching as low as 11.1% ± 1.0% and 13.0% ± 3.8%, respectively, by day 30 [9]​. Monitoring revealed that the viability of untreated control cultures remained high, illustrating the potential of Compound 15 as an effective therapeutic agent against GB by targeting and disrupting cell-cell adhesion mechanisms critical for tumor growth and maintenance ​[9]​.

Targeted therapies for glioblastoma stem cells

Targeted therapies for GB show promise, particularly those focusing on glioblastoma stem cells (GSCs). Despite multimodal treatments including surgery, chemotherapy, and radiotherapy, median survival for GB patients is approximately 14 months [10]​. The study established tumorspheres from U87MG GB cells, with the P4E8 clone displaying characteristics such as self-renewal, drug resistance, and high tumorigenic potential, indicating their utility as a GSC model [10]. P4E8 cells demonstrated resistance to several anticancer agents like cisplatin and etoposide, attributed to the overexpression of avidin-biotin-peroxidase complex transporters, suggesting potential targets for overcoming drug resistance​ [10]​. For monitoring and management, tamoxifen showed significant cytotoxic activity against P4E8 cells, highlighting its potential as a GSC-targeted therapy ​[10]​.

Metabolic pathway inhibitors

Some emerging therapies for GB focus on targeting the genetic and metabolic alterations characteristic of this highly aggressive cancer. Notably, inhibitors targeting metabolic pathways such as glycolysis, lipid metabolism, and glutaminolysis have shown promise. For instance, inhibitors of IDH1/2 mutations like AG-881 have advanced to phase III clinical trials, showing efficacy in reducing 2-HG levels and disrupting tumor growth [11]. Additionally, epidermal growth factor receptor (EGFR) inhibitors and tyrosine kinase inhibitors are extensively used to manage GB with EGFR amplifications and mutations ​[11]​. Monitoring and management of GB treatment include evaluating therapeutic response through genetic and metabolic profiling, ensuring personalized treatment strategies. Moreover, drugs like CB-839, a glutaminase inhibitor, are under clinical evaluation for their combined use with radiotherapy and temozolomide (TMZ) to enhance treatment efficacy ​[11]​.

Nanoparticle-based therapies

Nanoparticle-based therapies offer a promising approach for GB treatment by improving targeting and imaging of tumor cells. Silica-coated gold nanoparticles (AuNP-SHINs) were used to target GB multicellular tumor spheroids derived from U87-MG cells [12]​. These nanoparticles, functionalized with a tenascin-C antibody, demonstrated selective uptake primarily in the outer proliferating layer of the spheroids [12]​. However, they faced challenges penetrating deeper into the spheroid core, which became hypoxic and quiescent due to limited oxygen and nutrient availability. This restricted penetration was attributed to the size of the nanoparticles (77-78 nm), as smaller nanoparticles (<30 nm) have been shown to penetrate deeper into spheroids [12]. Monitoring revealed that despite the presence of tenascin-C in the core, nanoparticle uptake was limited to the outer 20-40 µm of the spheroids. The management of nanoparticle therapy should consider optimizing nanoparticle size and enhancing penetration strategies to overcome the barriers posed by tumor microenvironments, ensuring effective delivery and therapeutic outcomes [12].

Combination: metabolic inhibitors and traditional therapies

Combining metabolic inhibitors with traditional therapies shows potential in treating GB. A study highlighted the metabolic heterogeneity of GB, which complicates treatment due to varying resistance levels [13]. Notably, rapamycin (RAPA) combinations with metabolic inhibitors like doxycycline (DOXY) and etomoxir (ETO) significantly inhibited cell proliferation, with RAPA + DOXY reducing cell viability to 66% and RAPA + ETO to 70% [13]. These combinations target multiple metabolic pathways, enhancing therapeutic efficacy. Effective monitoring and management should involve mapping metabolic enzyme expression and activity using markers such as p-mTOR, p-S6, Rictor, p-Akt, CPT1A, and lactate dehydrogenase-A (LDHA) to guide personalized therapy and overcome resistance mechanisms ​[13]​.

PI3K/mTOR pathway inhibitors

The search for novel therapies to treat GB has identified promising compounds that target the PI3K/mTOR pathway, displaying significant antiproliferative activities. In a study, eCF324 emerged as the most potent compound, with EC50 (half-maximal effective concentration) values of 13 nM and 10 nM in U87 and T98 glioma cell lines, respectively, outperforming controls like INK128 and SN-38 [14]. Synergy studies revealed that combining eCF324 with the pan-PI3K inhibitor GDC0941 resulted in strong synergistic effects, particularly in T98 cells at concentrations of 100 nM eCF309 and 300 nM GDC0941. In patient-derived G317 glioma cells, eCF324 demonstrated a GI50 value of 7.2 nM, indicating superior potency compared to other inhibitors [14]. These findings support the potential of dual PI3K/mTOR inhibitors in GB therapy. Monitoring of therapeutic efficacy and safety showed that the tested compounds maintained high specificity for glioma cells, with minimal effects on non-cancerous cell lines at concentrations above 3 µM [14].

New tyrosine kinase inhibitor: K905-0266

The new tyrosine kinase inhibitor K905-0266 exhibits significant therapeutic potential in treating GB by targeting both GB cancer cells and cancer stem cells (CSCs) [15]. The compound effectively reduced cell viability in D54 and U87MG glioma cells, with bioluminescence imaging showing a reduction in efflux activity to 40.92% and 14.40% at 24 and 48 hours, respectively, in D54 cells, and to 68.15% and 21.33% in U87MG cells [15]. Additionally, K905-0266 treatment increased cleaved caspase-3 levels by up to 2.83-fold and decreased B-cell lymphoma 2 (Bcl-2) levels by up to 0.28-fold, indicating enhanced apoptosis [15]. Furthermore, K905-0266 reduced the protein kinase R-like endoplasmic reticulum kinase (pERK) levels by up to 0.11-fold, inhibiting the mitogen-activated protein kinase (MAPK) pathway. In CSCs, K905-0266 significantly decreased sphere formation, indicating its efficacy against therapy-resistant CSCs [15]. Monitoring and management were conducted using bioluminescent imaging and Western blot analyses, confirming the compound's inhibitory effects and ensuring precise tracking of therapeutic efficacy and safety​ [15]​.

Adenovirus Ad5-Ki67/IL-15

A novel double-controlled oncolytic adenovirus driven by the Ki67 core promoter and armed with interleukin 15 (IL-15) demonstrates significant potential for treating GB by selectively infecting and killing GB cells while sparing normal cells. The adenovirus Ad5-Ki67/IL-15 significantly increased IL-15 expression in treated GB cells, with IL-15 concentrations reaching 10.325, 12.97, and 19.32 pg/ml in GL261, U251, and U87 cells, respectively, after 96 hours, and 9.71 pg/ml in BT-01 cells after 72 hours [16]. This enhanced antitumor effect was further confirmed by a marked reduction in cell viability, which was significantly greater in Ad5-Ki67/IL-15-treated cells compared to controls. Additionally, Ad5-Ki67/IL-15 showed strong anti-angiogenic properties by decreasing vascular endothelial growth factor (VEGF) secretion, which is crucial for angiogenesis in GB [16]. Monitoring and management involved using enzyme-linked immunosorbent assay (ELISA) to measure interleukin 15 (IL-15) and vascular endothelial growth factor (VEGF) levels and fluorescence microscopy to observe virus infection efficiency, ensuring accurate tracking of therapeutic efficacy and safety [16].

2-Aminothiazole-flavonoid hybrid compounds

A new series of seventeen 2-aminothiazole-fused flavonoid hybrid compounds demonstrates significant potential in treating GB by targeting Tau protein, which is overexpressed in various brain cancers including GB. Compounds 2 and 9 showed high affinity for Tau, inhibiting Tau fibrillation and displaying strong anti-metabolic activity in several Tau-expressing GB cells. Specifically, these compounds increased mitochondrial network fission and induced microtubule bundling within newly formed neurite-like protrusions, leading to reduced cell migration. The monitoring and management of these therapies involve assessing Tau binding, fibrillation inhibition, and cellular effects on Tau-expressing cells, ensuring precise tracking of therapeutic efficacy and safety [17]​.

CD70-directed CAR-T cells

Therapies targeting GB, particularly through immunotherapeutic approaches like CD70-directed CAR-T cells, show promising results in overcoming therapy resistance and improving patient outcomes. CD70 expression, elevated in recurrent GB, plays a crucial role in tumor aggressiveness and maintenance. CD70 knockdown in GB cells significantly reduces tumorigenicity in vitro and in vivo, and CD70 CAR-T therapy enhances survival rates in animal models, indicating a robust immune response against CD70-expressing GBs [18]. Monitoring the tumor immune microenvironment (TIME) is vital as CD27, the receptor for CD70, is found on relevant GB TIME cell populations ​[18], suggesting the potential of co-targeting GB and its microenvironment for improved therapeutic outcomes. The presence of CD70 in GB but not in normal tissues supports its suitability as a therapeutic target, with CD70-directed CAR-T cells displaying significant cytotoxicity and decreased tumor growth in preclinical models [18].

Radiotherapy and TMZ

Therapies for the treatment of GB show limited efficacy due to the tumor's recurrence and resistance. Standard treatments like surgical resection followed by radiotherapy and temozolomide (TMZ) lead to a median survival of only about 14 months, with local relapse being inevitable. Transcriptome profiling of patient-matched initial and recurrent GB samples revealed 147 significant probes, with 24 validated genes involved in angiogenesis and immune-related processes [19]. Notably, genes like SDC2 and CXCL12 were upregulated, indicating potential targets for immunotherapy [19]. Drug repurposing identified Food and Drug Administration-approved drugs such as rosiglitazone, nizatidine, pantoprazole, and tolmetin as promising therapies against GSCs and GB recurrence [19]. Effective monitoring and management of GB require longitudinal transcriptional profiling to adapt treatment strategies to the evolving tumor biology, emphasizing the necessity for personalized therapeutic approaches [19]​.

P2Y12 receptor target

Therapies for GB involve targeting the P2Y12 receptor, which has shown promise in reducing tumor proliferation and migration. Treatment with the P2Y12 receptor antagonist ticagrelor significantly decreased cell viability and proliferation in both the U251 and LS12 GB cell lines, with a reduction in cell viability by approximately 34.2% in U251 and 44.2% in LS12 [20]. The clonogenic assay further supported these findings, with a notable reduction in colony formation upon ticagrelor treatment. Additionally, ticagrelor treatment reduced adenosine diphosphate hydrolysis activity in U251 cells and increased autophagy, as indicated by acridine orange staining [20]. Monitoring and managing GB treatment effectiveness involves assessing changes in molecular markers and cellular responses to therapies, such as alterations in cell viability, proliferation, migration, and induction of autophagy, to optimize and personalize therapeutic strategies ​[20].

Surgical resection followed by radiation and temozolomide

Therapies for the treatment of GB often face significant challenges due to the tumor's recurrence and resistance to standard treatments. Standard treatment includes surgical resection followed by radiation and temozolomide, but the median survival remains around 15 months. Recent studies show that blocking Ig-like transcript 2 (ILT2), an inhibitory checkpoint receptor, can restore antitumor immune responses. ILT2 blockade increased natural killer cell-mediated cytotoxicity and interferon-gamma (IFN-γ) production, enhancing the immune response against GB cells ​[21]. Combining ILT2 blockade with temozolomide further increased tumor cell elimination, demonstrating a synergistic effect. Monitoring and management of GB therapies require regular assessments of immune cell activity and tumor response to adapt treatment strategies accordingly ​​[21].

Anti-O-acetyl GD2 (OAcGD2) monoclonal antibody 8B6 with TMZ

The combination of anti-O-acetyl GD2 (OAcGD2) monoclonal antibody 8B6 with TMZ enhances the treatment efficacy against GB by targeting GSCs that drive chemoresistance. A study demonstrated that GSCs express high levels of OAcGD2, and the use of 8B6 increases TMZ genotoxicity and tumor cell death in vitro [22], reducing the expression of GSC markers CD133 and Nestin ​[22]. In vivo, the combination therapy significantly inhibits tumor growth and reduces the GSC pool compared to monotherapy ​[22]. Specifically, the combination therapy resulted in a tumor volume reduction to 26.8 ± 12.8 mm³, significantly lower than TMZ alone (265.2 ± 26.8 mm³) and 8B6 alone (132.7 ± 27.1 mm³) [22]. Monitoring through flow cytometry and limiting dilution assays confirmed that the combination regimen effectively impaired GSC's self-renewal capabilities. This synergistic approach offers a promising strategy for overcoming TMZ resistance and improving GB management ​[22]​.

Dendritic cell (DC) vaccines targeting cytomegalovirus (CMV) antigens

Combination dendritic cell (DC) vaccines targeting cytomegalovirus (CMV) antigens offer a promising approach for GB treatment, enhancing survival rates compared to standard therapies. In three sequential clinical trials, patients treated with CMV-specific DC vaccines showed significantly improved outcomes, with 33-36% overall survival (OS) rates at five years. For instance, in the ATTAC-GM trial, the median overall survival (mOS) was 37.7 months [23], surpassing the typical 14.6-20.9 months observed with conventional treatments. Monitoring included assessing DC migration to lymph nodes and patient survival rates. Patients receiving GM-CSF-containing DC vaccines with dose-intensified temozolomide exhibited prolonged survival, with 55% remaining tumor-free at 40 months and 36% at five years​ [23]. These findings underscore the potential of DC vaccines in extending survival and managing GB, highlighting the importance of personalized immunotherapy strategies in combating this aggressive cancer.

Veliparib, TMZ, and radiotherapy

Combination therapies integrating veliparib with TMZ and radiotherapy show limited improvement in treating GB with unmethylated O-6-methylguanine-DNA methyltransferase promoter status. In the veliparib, radiotherapy, and temozolomide (VERTU) study, the experimental arm receiving veliparib in combination with radiotherapy followed by TMZ did not significantly enhance progression-free survival (PFS) at six months compared to the standard TMZ and radiotherapy regimen, with PFS-6m rates of 46% and 31%, respectively [24]. mOS was similar between both groups: 12.7 months for the experimental arm and 12.8 months for the standard arm. Monitoring included regular magnetic resonance imaging (MRI) scans to assess tumor progression and frequent blood tests were conducted to monitor toxicity, particularly for adverse events like thrombocytopenia and neutropenia. These findings indicate that while veliparib is safe and tolerable, it does not provide substantial clinical benefit over existing treatments for this specific patient population [24].

Lysate-loaded dendritic cell vaccination (DCVax-L)

Lysate-loaded dendritic cell vaccination (DCVax-L), an autologous tumor lysate-loaded dendritic cell vaccine, significantly extends survival in GB patients. In a phase 3 trial, mOS for the newly diagnosed GB patients receiving DCVax-L was 19.3 months from randomization compared to 16.5 months for the control group (hazard ratio (HR) = 0.80; P = .002) ​[25]​. For recurrent GB patients, mOS was 13.2 months versus 7.8 months in the control group (HR = 0.58; P < .001). At 60 months, 13.0% of newly diagnosed patients treated with DCVax-L were alive compared to 5.7% in the control group ​[25]​. Monitoring included regular MRI scans and survival analysis, and management involved potential additional treatments post-recurrence, such as bevacizumab and lomustine. DCVax-L showed a favorable safety profile with minimal serious adverse events [25]​.

Difluoromethylornithine (DFMO) and AMXT-1501

Therapies for the treatment of GB show potential improvement with the combination of difluoromethylornithine (DFMO) and AMXT-1501. The Phase 0 trial evaluated the impact of polyamine depletion and polyamine transport inhibition on the extracellular metabolome within high-grade gliomas [26]​. The initial results indicate that DFMO, combined with AMXT-1501, significantly alters intratumoral metabolite levels, with guanidinoacetate showing a 126-fold increase in tumors compared to adjacent brain tissue, and polyamine depletion leading to increased glutamate levels [26], a marker of cytotoxicity. Monitoring through microdialysis catheters enables real-time assessment of drug efficacy and metabolic changes postoperatively, ensuring precise therapeutic interventions and minimizing adverse effects. The trial underscores the importance of targeted metabolic therapies and their monitoring to manage GB effectively ​[26].

Kinase inhibitors and apoptosis pathway inducers

The integration of multi-targeted therapies has shown promise in the treatment of GB, highlighting significant synergistic effects. Notably, the combination of kinase inhibitors and apoptosis pathway inducers, such as lapatinib with obatoclax mesylate, demonstrated strong efficacy in patient-derived GB cultures. A study identified 15 out of 43 drug combinations with consistent synergistic interactions, with 11 combinations showing long-term effects [27]​. Specifically, combining Venetoclax (BCL targeting) and AZD5991 (MCL1 targeting) yielded high synergy scores, indicating a potent strategy for GB treatment ​[27]. Monitoring and management of these therapies are crucial due to potential toxicities; for example, MCL-1 inhibitors can cause cardiovascular issues, necessitating close health monitoring during combination treatments ​[27]​.

The Voyager system

The Voyager system is a safe and feasible treatment option for recurrent GB, showing promising results in improving patient outcomes. In a study involving 75 patients, the median PFS was 17 weeks for those treated with Voyager alone and 21 weeks for those treated with Voyager combined with standard chemotherapy [28]. The mOS was seven months for Voyager alone and nine months for the combined treatment group [28]. Notably, patients in their first or second recurrence had a median OS of 10 months with the combined treatment. The Voyager system, which uses an ultra-low radio frequency energy signal, had no serious device-related adverse events. Monitoring included MRI scans every 42 days and the most common side effects were fatigue and headaches, which were manageable​ [28].

Personalized peptide vaccination (PPV)

Personalized peptide vaccination (PPV) for the treatment of recurrent GB showed limited effectiveness in improving OS in a phase III trial [29]. The trial included 88 patients, randomly assigned to receive either PPV (n=58) or placebo (n=30). The median OS was 8.4 months for the PPV group and 8.0 months for the placebo group, failing to meet the primary endpoint [29]. Secondary endpoints, including PFS and one-year survival rates, also showed no significant differences [29]. PPV patients without SART2-93 peptide selection who were under 70 years old or weighed less than 70 kg had better outcomes, with a median OS of 9.6 months compared to 4.7 months for corresponding placebo patients [29]. Monitoring included regular MRI scans and assessments of immune responses. Despite some immune activation, the study concluded that PPV might not be beneficial for all the patients and highlighted the need for further investigation into more effective treatments ​[29]​.

Oncolytic viral therapy (OVT)

Oncolytic viral therapy (OVT) presents a promising novel approach for treating GB. In recent clinical trials, OVT has demonstrated the potential to enhance OS and PFS in patients with recurrent GB [30]. Among the 29 trials analyzed, 18 were phase I, highlighting the early stages of this research [30]. The median PFS reported was three months, and the median OS was 15 months after the first viral dose. The most commonly used viral vectors included adenovirus and herpes simplex virus, targeting tumor cells through various mechanisms such as direct lysis, immune response activation, and inhibition of tumor vascularization [30]. Monitoring and management involved regular MRI scans, physical exams, and laboratory tests to track treatment efficacy and patient safety. Despite limited results, the ongoing trials indicate a significant potential for OVT to be integrated into standard GB treatment protocols once safety and efficacy are fully established ​[30]​.

Bone Morphogenetic Protein 4 (BMP4)

Bone Morphogenetic Protein 4 (BMP4) significantly impacts GB treatment by inducing asymmetric cell division (ACD) and reducing the self-renewal capacity of GSCs. In the study, BMP4 treatment decreased the expression of CD133, a marker of CSCs, and increased the ACD ratio from 23% to 38% (P=0.004) [31]​. The sphere-forming ability of GSCs was notably reduced under BMP4 treatment, demonstrating a significant suppression of self-renewal capacity [31]​. BMP4 also inhibited cell proliferation and promoted neural differentiation markers without affecting cell viability [31]​. This differentiation therapy approach suggests BMP4 could enhance the effectiveness of conventional chemotherapy and radiation therapy by reversing drug resistance and reducing the tumorigenic potential of GSCs. Effective monitoring and management of GB involve targeting the BMP4-Smads signaling pathway, reducing CD133-positive cells, and employing combination treatments to enhance therapeutic efficacy ​[31].

Tumor-treating fields (TTFs)

TTF therapy for GB shows a mixed impact on patient outcomes and quality of life (QoL). In a study of 30 high-grade glioma patients, the mean overall QoL score for those treated with TTFds was 50.3 compared to 45.7 in the control group, though not statistically significant ​[32]​. Patients using TTFds reported better emotional functioning and a lower incidence of insomnia and appetite loss. However, 71% of patients experienced moderate to very severe restrictions in daily activities due to the therapy, particularly in exercise, body care, and social relationships ​[32]. Despite these restrictions, 67% of patients expressed willingness to reuse TTFds based on their experience. Effective monitoring and management involved the use of standardized questionnaires (EORTC QLQ-C30, QLQ-BN20, QLQ-FA13) to regularly assess the physical, emotional, and cognitive functions, as well as the symptom burden and social support of patients during treatment [32]​.

Limitations of the study

The study, while comprehensive, has some limitations that should be considered when interpreting the findings. First, the inclusion of only English-language articles may have introduced a language bias, potentially excluding relevant studies published in other languages. Additionally, the review focused solely on peer-reviewed journal articles, which could overlook valuable data presented in non-peer-reviewed sources such as conference abstracts or gray literature. The heterogeneity among the included studies, particularly regarding sample sizes, study designs, and treatment protocols, poses challenges for direct comparison and synthesis of results. Furthermore, the reliance on preclinical models for evaluating emerging therapies limits the generalizability of some findings to clinical practice, as many promising preclinical results do not translate into clinical success. Finally, the evolving nature of glioblastoma treatment means that the rapidly changing therapeutic landscape could render some of the findings outdated, necessitating continuous updates and validation of the review's conclusions.

## Conclusions

Emerging therapies for glioblastoma (GB) show significant promise, with several innovative approaches demonstrating efficacy. Based on our findings in the review, targeted therapies focusing on GSCs, such as tamoxifen, have shown cytotoxic activity. Nanoparticle-based therapies and combination treatments, including kinase inhibitors and apoptosis inducers, enhance targeting and therapeutic efficacy. Immunotherapeutic strategies, including CD70-directed CAR-T cells, and personalized peptide vaccinations, demonstrate potential for overcoming resistance and improving patient outcomes. Bone Morphogenetic Protein 4 and oncolytic viral therapy further highlight the diverse approaches being explored to tackle this aggressive cancer, with research necessary to optimize these promising strategies.
